# Intestinal microbiota-mediated dietary fiber bioavailability

**DOI:** 10.3389/fnut.2022.1003571

**Published:** 2022-10-28

**Authors:** Kangxiao Guo, Zihan Yao, Tao Yang

**Affiliations:** ^1^National Engineering Laboratory for Rice and By-Product Deep Processing, College of Food Science and Engineering, Central South University of Forestry and Technology, Changsha, China; ^2^Pharmacy Department, Changsha Health Vocational College, Changsha, China

**Keywords:** dietary fiber, intestinal microbiota, short-chain fatty acids, biological availability, mediated

## Abstract

Dietary fiber is a kind of carbohydrate that cannot be digested and absorbed by the small intestine of humans but can be fermented in all or part of the large intestine and is significantly healthy for the human body. With the improvement in living standards, people pay more attention to their intestinal health, and the relationship between dietary fiber, intestinal microecological and body physiological balances, and their molecular connection mechanism has become a research hot spot. In this study, we reviewed its mediated bioavailability to provide a basis for the rational classification of dietary fiber and to guide the development of new healthy foods and the deep processing of food and its application.

## Introduction

In 1941, Duck Worth and Godden (1) coined the term “dietary fiber” to study the effects of rat feed-added pulp on intestinal mucus secretion. In 1953, the term was first directly referred to by Hipsley (2) as a high-fiber diet that reduced the incidence of “pregnancy toxicosis”. In the 1860s and 1880s, the high-fiber diet (3–7) was found to prevent diseases of modern civilization and colorectal bacteria *via* a rumen-like fermentation function. These are the two major milestone events in the history of modern scientific research on dietary fiber. Nowadays, with increasing attention paid to intestinal health, the balanced relationship between dietary fiber, intestinal microecology and body physiology, and its molecular connection mechanism has become a research hot spot. Most of the nutrients in the gut flora can be obtained from gastrointestinal secretions or metabolites between the bacteria, but the main carbon source for the growth and metabolism of the gut bacteria can only be obtained from the fiber (8–10). In the current study, we reviewed the effects of dietary fiber on intestinal microecology to provide a basis for the rational classification of dietary fiber and to guide the development of new healthy food and the deep processing of food and its application.

## Bioavailability of dietary fiber

**Figure 1:**
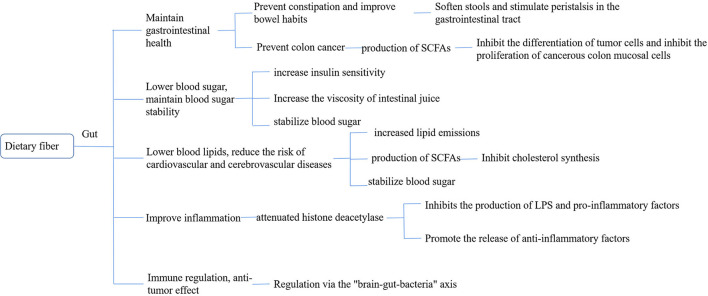
Bioavailability of dietary fiber.

Dietary fiber (DF) is a class of carbohydrates (11) present in plant foods that cannot be digested and absorbed by the small intestine of humans but can be completely or partially fermented in the large intestine. Dietary fiber mainly includes (12) cellulose, hemicellulose, pectin, and inulin. In recent years, wax, cuticle and undigested cell wall proteins, resistant starch, Maillard reaction products, and animal-derived digestive substances have been classified as dietary fibers (13). Dietary fiber is known as the seventh major nutrient because of its physiological functions such as lowering of the lipid levels, regulation of sugar metabolism, regulation of intestinal microbes, and reduction of the risk of depression. Unlike protein, which can directly participate in the regulation of physiological function, dietary fiber is mainly directly or indirectly used by intestinal flora through its special structure, affecting the structure of intestinal flora and its metabolites (14) and then regulating the physiological function of the body through the brain–intestinal axis (Figure 1).

### DF maintains gastrointestinal health

#### Prevention and treatment of constipation and improvement of defecation habits

Dietary fiber expands the human digestive tract through water absorption, increasing stool volume, softening stool, stimulating gastrointestinal peristalsis, and relieving constipation. Castillejo (15) divided the patients with long-term constipation with two groups A, B. Group A children were provided with dietary fiber supplements, and group B with placebo, and both the groups underwent a double-blind test. After 4 weeks, compared to children in group B, children in group A showed significantly reduced colon transmission time, increased fecal volume, and significantly higher defecation. The present study confirms that supplementing with dietary fiber can speed up intestinal peristalsis, relieve constipation, and improve stool habits. Dahl et al. (16) conducted a study on 114 elderly people for 6 weeks and found that adding 1–3 g of dietary fiber increased the average number of bowel movements by 2–3 times per month and from 8.8 ± 1.0 times to 12.6 ± 3.8 times in patients with severe constipation.

#### Prevention and treatment of colorectal cancer

Colorectal cancer (CRC), a malignant tumor, has a high incidence and mortality rate and is one of the most common types of cancer (17). In recent years, the incidence of CRC in China has continued to increase and has become highly prevalent. Ezzie's “second-hit doctrine” suggests that the key factor in susceptibility to tumorigenesis is the host (18). Binghamsa et al. (19) assessed the risk of CRC in patients with different DF intakes, found a relative risk (RR) of 0.58 (95% CI: 0.41–0.85) in the highest DF group and the lowest intake group, and a 40% reduction in the risk of CRC in those with high DF intake, indicating a linear relationship between DF intake and CRC risk. Accumulating evidence reported that gut microbiota could play important roles in the occurrence and progression of colorectal cancer. Dietary fibers are fermented by the intestinal microbiota. Jujube fruit, which predominantly contains polysaccharides, has been shown to inhibit carcinogenesis in animal models. The intake of large amounts of DF can effectively prevent CRC and other chronic diseases; however, the reason behind this finding is still elusive (20). Currently, the mainstream hypothesis is that dietary fiber is converted into short-chain fatty acids (SCFAs) through the decomposition of intestinal flora to prevent and control colon cancer (21). SCFAs, an important fermentation product in DF, play a crucial role in intestinal health (22). Acetate is one of the energy sources of human colon epithelial cells, muscles, and brain tissue. It can reduce the pH of pathogens in the body by reducing the solubility of bile salts, increasing the absorption of minerals, and reducing the absorption of ammonium (23, 24) propionate, which not only reduces the amount of cholesterol in colon pH and blood but also reduces hepatic adipogenesis. Inhibition of CRC cell proliferation, induced cell apoptosis, and also enabled increased colonic blood flow and oxygen uptake, which had anti-inflammatory effects, which is also the effects of propionate. Butyrate can provide energy to human small intestinal epithelial cells, inhibit tumor cell differentiation, inhibit the proliferation of cancerous colon mucosal cells, and play an anti-cancer role (25, 26).

### DF lowers blood sugar and maintains blood sugar stability

After the consumption of food through the mouth, it is initially digested in the stomach and then enters the small intestine. The digestive enzymes in the small intestine digest, decompose, absorb, and utilize it, increasing the blood sugar level and reaching its peak. Dietary fiber becomes sticky after the absorption of water, which hinders the decomposition and utilization of food, thus inhibiting glucose absorption and utilization, increasing gastric emptying time, and stabilizing the blood glucose level (27). Therefore, adequate intake of dietary fiber reduces postprandial blood glucose levels and thus reduces risk factors for chronic diseases such as glucose intolerance, hyperinsulinemia, and postprandial hyperlipidemia. A large number of studies showed that dietary fiber can reduce postprandial blood glucose levels (28, 29), which may be related to its mechanism of inhibiting beta-amylase activity during adsorption, thereby delaying glucose diffusion and digestion. Benitez et al. (30) found that the ability of coffee parchment, a non-water-soluble dietary fiber, to adsorb glucose increased with the increase in glucose concentration and that coffee parchment inhibited the diffusion of glucose to some extent *in vitro*. The inhibitory mechanism of glucose utilization by coffee parchment is similar to that of cellulose, which inhibits beta-amylase activity, prolongs the carbohydrate digestion time, reduces the glucose absorption rate, and prevents increases in blood glucose. Kay et al. (31) demonstrated by *in vitro* simulated small intestinal digestion experiments that soluble dietary fiber in the pudding matrix can produce viscous substances in the small intestine, prevent glucose absorption, and reduce the postprandial blood glucose peak and insulin. Khaled et al. (32) studied the effect of pectin on amylase activity in mice under high-lipid and high-glucose conditions and found that pectin could significantly inhibit beta-amylase activity and lead to a 24% decrease in blood glucose levels.

### DF lowers blood lipid and reduces the risk of cardiovascular and cerebrovascular diseases

Hyperlipidemia can cause a series of cardiovascular and cerebrovascular diseases, such as coronary heart disease, cerebral infarction, and atherosclerosis. Increasing the intake of viscous and soluble dietary fiber has been recommended as a safe and practical method to reduce cholesterol (33). Li et al. confirmed that the dietary fiber in *Dendrobium officinale* presented varying degrees of inhibition/promotion of TG, TC, LDL-C, and HDL-C (34). SCFAs are absorbed by the portal vein after the fermentation of the dietary fiber generated by the intestinal microorganisms, limiting the activity of 3-hydroxy-3-methyl glutaryl-CoA reductase (3-hydroxy-3-methylglutaryl-CoA, HMG-CoA) and increasing the catabolism of LDL-C, which inhibits the synthesis of liver cholesterol (35). Dietary fiber in the water membrane of the digestive tract creates a highly viscous environment, which not only hinders the reabsorption of fat, cholesterol, and bile acid by the digestive tract but also increases insulin sensitivity, increases satiety, changes the circulating level of bile acid, increases the production of therapeutic ursodeoxycholic acid, inhibits the absorption of toxic choleric acid, and reduces the possibility of cholesterol absorption (36). By binding to bile acids in the gut, dietary fiber reduces its reabsorption, increases excretion, and lowers bile acid levels. To compensate for this loss, the body synthesizes bile acids *de novo* and activates CYP7A1, which increases the synthesis of bile acids and the fecal excretion of neutral sterols and cholesterol and reduces the cholesterol concentration. Dietary fiber promotes the *de novo* synthesis of bile acids, upregulates LDL-C receptors, provides a substrate for bile acid synthesis, increases LDL-C removal, and decreases the LDL-C concentration (37).

### DF improves inflammation

Jesenak et al. (38), by performing oat–glucan intervention after intraperitoneal LPS injection in a mouse model, found that beta-glucan could increase the amount of GLP-2 in mouse plasma, while GLP-2 enhanced the proliferation of the intestinal epithelium and reduced intestinal permeability, thereby reducing lipopolysaccharide absorption and inhibiting the formation of non-alcoholic hepatitis. Fei et al. provided their study participants with a WTP diet of whole grains, traditional Chinese medicine, and prebiotics, and 23 weeks later, they found that the intestinal microecological environment improved, the intestinal flora became balanced, inflammation levels decreased (39), and hypertension and hyperglycemia symptoms were significantly relieved. SCFAs produced by the fermentation of dietary fiber play an important role in regulating the occurrence of inflammatory responses and can intervene in the production of inflammatory factors by inhibiting histone deacetylase (HDAC) (40). SCFAs, which not only can inhibit the production of proinflammatory factors, but also can promote the release of anti-inflammatory factors. Many G protein-coupled receptors (GPRs), which are expressed on the surface of inflammatory cells, can regulate the activity of transcription factors and affect the synthesis and secretion of inflammatory factors. When SCFAs act on GPRs, they can quickly activate the intracellular pathways, including mitogen-activated protein kinase, protein kinase c, and transcription factors, thus reducing the synthesis and secretion of pro-inflammatory factors, reactive oxygen clusters, cyclooxygenase-2, etc., and inhibiting the inflammatory response (41). SCFAs can also inhibit the acetylation of multiple proteins in the NF-B signaling pathway family through HDAC, weaken the body LPS response mechanism, inhibit the phosphorylation of IKB, and thus inhibit the activation of the NF-B pathway (42).

### Immunomodulatory and anti-tumor effects

Recently, studies demonstrated that SCFAs play an important role in maintaining intestinal immune responses and individual immune responses. SCFAs can serve not only as energy substances but also as signaling molecules. Their production is closely related to dietary fiber in the ingested food. Intestinal SCFAs are produced by DF that cannot be decomposed in food, cannot be decomposed by the body, and are fermented by microorganisms in the gut. SCFAs not only provide enough energy for the microorganisms in the gut but also have an important role in the individual immune system (43). When dietary fiber in food is insufficient, the fermentation activity of gut microbes decreases, and the content of SCFAs also decreases. Park et al. (44) found that SCFAs can directly affect T-cell differentiation and promote differentiation of T cells into effector T cells, such as Th1 and Th17, thereby promoting T-cell differentiation and cytokine expression through the activation of GPR41- or GPR43-dependent HDAC inhibitors and the subsequent activation of mTOR-S6K. SCFAs can act through the “brain–gut axis” and affect the host body state (45). For example, butyrate can change the expression of many different functional genes by inhibiting histone deacetylase (HDAC) and controlling cell differentiation and proliferation of apoptotic genes, thus inhibiting colorectal inflammation and colorectal cancer. In cancer cells, when butyrate is concentrated three times higher than that in normal cells, it can act as an inhibitor of HDAC and effectively inhibit its activation (21).

## Relationship between dietary fiber and intestinal microflora

During human evolution, the supply of energy to the body changed with the environment and time, and the type and quantity of carbohydrates consumed also changed daily. However, humans and most animals do not digest some carbohydrate enzymes; for the human genome, change or restructuring is impossible, and intestinal flora needs to have the ability to adjust its metabolism to adapt to the change. Therefore, the metabolism of complex carbohydrate tasks to intestinal microbes, is a very effective and flexible way, which can help human to adapt to the complex diet environment (46). The degradation of dietary fiber is controlled by the carbohydrate-active enzymes (CAZymes), which encode only 17 enzymes in the human genome involved in carbohydrate digestion (eight of which have known functions) (47), which are mainly responsible for the degradation of non-resistant starch, lactose, maltose, maltose oligosaccharides, sucrose, and trehalose. The intestinal flora can use a large class of carbohydrate-active enzymes that can hydrolyze various complex glycans to catalyze or transform dietary fiber accordingly. The resulting metabolites can not only provide energy for their growth but also improve the biological function of the host. Intestinal flora-encoded carbohydrate-active enzymes can cleave sugar bonds between monomers or between carbohydrate and non-carbohydrate structures, according to their amino acid sequence. Carbohydrate-active enzymes are divided into four categories (Table 1): glycoside hydrolase (GHs), polypyrolyase (PLs), carbohydrate esterase (CEs), and auxiliary enzymes (48). The most important fermentation products of dietary fiber in the cecum and colon are SCFAs; among them, butyrate is the most abundant compound present in the colon and the cecum. The diet structure and fiber type can affect the production of short-chain fatty acids by regulating intestinal microbes. SCFAs also greatly impact body organs and metabolism.

**Table 1:** Function of four classes of carbohydrate-active enzymes encoded by intestinal flora.

**Enzym**	**Function**
GHs	Cut the glycosidic bonds between 2 or more sugar units or between the sugar and non-sugar moieties
PLs	It catalyzes the breakage of the acidic sugar unit (i.e., glucuronic acid and galacturonic acid) contained in the polysaccharide chain
CEs	Hydrolyze possible ester bonds in pectin (pectin methyl ester) and acetylating arabinoxylan
Auxiliary enzyme	Degradation of lignin

## Mechanisms by which intestinal flora mediate the bio-effectiveness of dietary fiber

Dietary fiber achieves its bio-effectiveness mainly by influencing the diversity, metabolites, and living environment of intestinal flora.

### Effect of dietary fiber on the diversity of the intestinal microflora

The balance of intestinal microecology is related to the health of the body, and the quantitative ratio of intestinal microbial colonization resistance, that is, the ratio of *Bifidobacterium* to *Enterobacter*, is used as a sign of the balance of intestinal flora. Dietary fiber provides a substrate for flora growth and metabolism, which is conducive to the increased diversity and/or abundance of intestinal flora. After feeding mice with a cellulose-rich diet, Berer et al. (49) found an increase in *Ruminococcaceae, Helicobacteraceae*, and *Enterococcaceae* and a decrease in the abundance of *Sutterellaceae, Lactobacillaceae*, and *Coriobacteriaceae*. Zhai et al. (50) also found that simultaneous supplementation of a mixture of insoluble fiber and soluble fiber could significantly increase the relative abundance and diversity of the gut microbiota, regulate fatty acid metabolism, and thus prevent obesity. Martinez et al. (51) studied the fecal microbial populations in mice fed with different types of resistant starch (RS); they found that RS4 at the phylum level could promote an increase in actinomyces and *Bacteroides*, which can reduce Firmicutes. At the species level, it can cause an increase in *Bifidobacterium adolescentis* and *Parabacteroides distasonis*; RS2 significantly increased the proportion of *Ruminococcus bromii* and *Eubacterium rectale*. They found that the number of *Actinobacteria* and *Bifidobacteria* in the gut of barley glucan-fed mice increased significantly. Miyamoto et al. (52) and Bai et al. (53) added bitter gourd powder to the diet of high-fat-induced obese rats and found that the number of warts microbacteria (Verrucomicrobia) and departmental butyrate bacteria (*Blautia* and *Allobaculum*) increased significantly. Barczynska et al. (54) showed that potato dextrin could increase the number of *Lactobacillus, Bifidobacterium, Prevotella*, and *Bacteroides* in the intestine of high-fat diet-induced obese rats and significantly inhibit the growth of *Clostridium*. Zhang et al. (55), using a diabetic rat model with inulin intervention, found that compared with normal control rats, the proportion of *Firmicutes* increased, the proportion of *Bacteroides* decreased, and rat intestinal flora characteristics in the control group and inulin treatment group were similar. In Zhang's study, lactobacillus and SCFAs flora in the treatment group than in the untreated group, indicating that inulin by promoting the growth of intestinal probiotics, improves diabetic rat intestinal flora structure, makes its homeostasis, and alleviates the politics of diabetes. Thus, it shows that dietary fiber increases the gut microflora by optimizing the structure of intestinal flora.

### Effect of dietary fiber on gut microbiota metabolites

**Figure 2:**
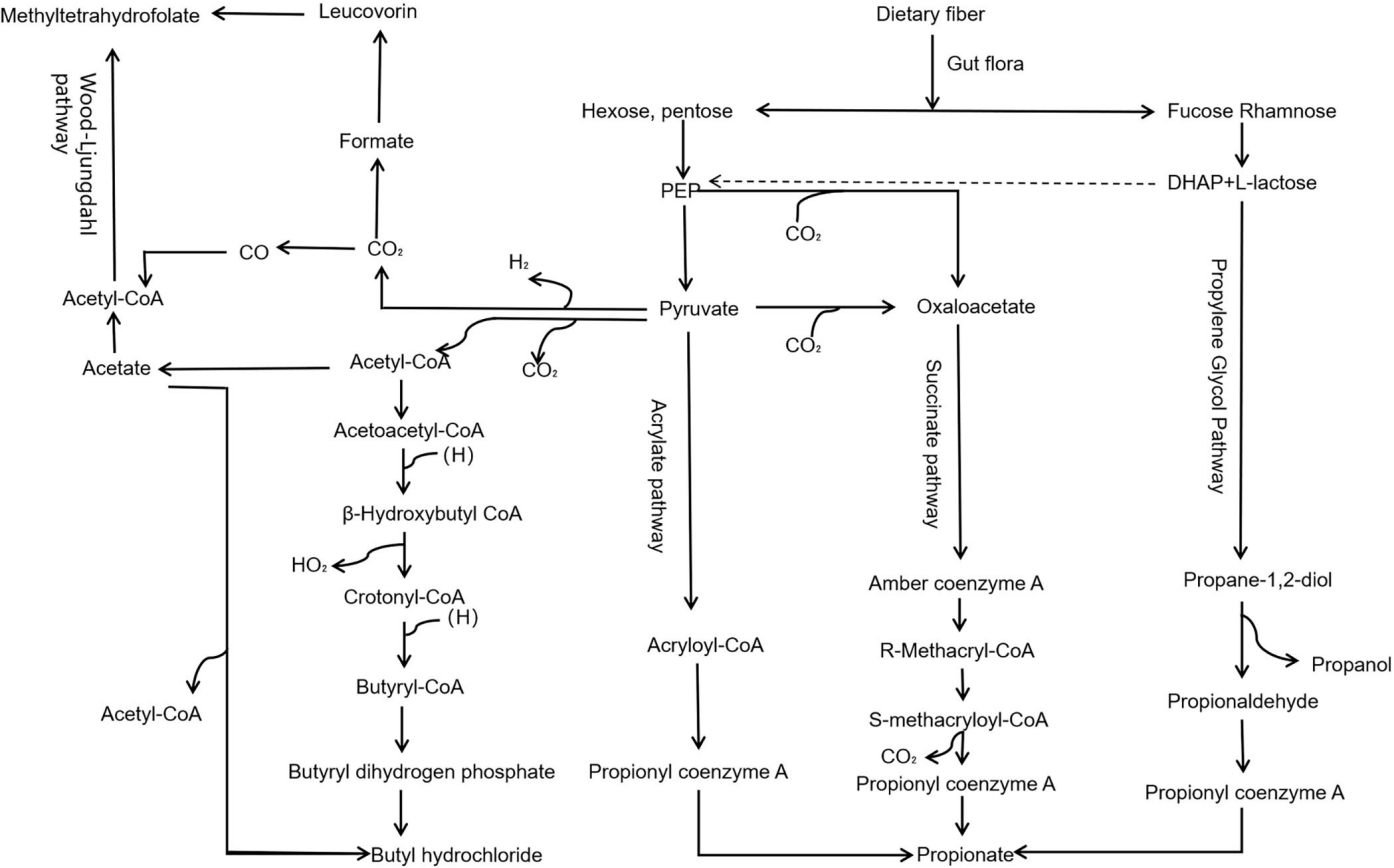
Pathway of dietary fiber generates SCFAs *via* gut microbiota. Dietary fiber in the gut, through propylene glycol, succinate, acrylate, and Wood–Ljungdahl pathway, generates important metabolites, such as SCFAs, including acetate, propionate, and butyrate.

**Figure 3:**
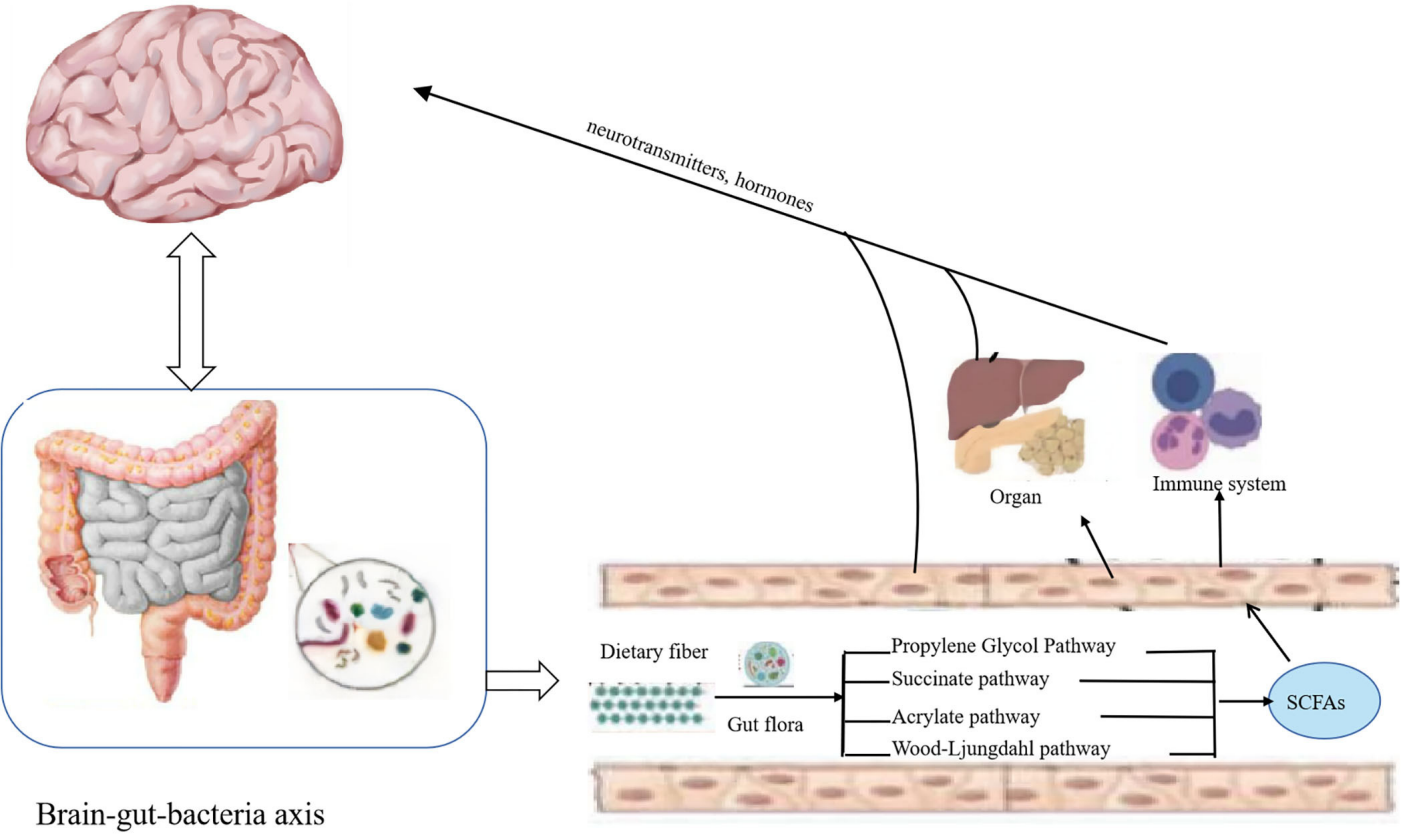
Dietary fiber exerts its bioavailability through the brain–gut–bacteria axis. Short-chain fatty acids produced by the degradation of dietary fiber enter the hepatocytes and transform into small metabolic molecules with regulatory activities, break the blood–brain barrier and enter the central entry system, and play a regulatory role in the body by affecting the brain–gut–bacteria axis.

Dietary fiber in the gut through propylene glycol, succinate, acrylate, and Wood–Ljungdahl pathway (Figure 2) generates important metabolites like SCFAs, including acetate, propionate, and butyrate. SCFAs play roles in intestinal cell absorption and utilization for the microbiota itself and body energy, affect intestinal flora growth, and protect the intestinal tract. However, SCFAs in the intestine without the colon cell metabolism of SCFAs through the basolateral membrane into the hepatic portal vein circulation, the oxidation provides an energy substrate for liver cells. In the biosynthesis of glucose, cholesterol, and fatty acids, short-chain fatty acids enter the hepatocytes and transform into small metabolic molecules showing regulatory activities, break the blood–brain barrier and enter the central entry system, and play a regulatory role in the body by affecting the brain–gut–bacteria axis (Figure 3) (56, 57). For example, the dietary fiber in ginseng, pumpkin, and *Angelica sinensis* produces a large amount of SCFAs. SCFAs can directly act on various signaling pathways, organs, and the immune system and regulate the release of total cholesterol (TC), triglyceride (TG), inflammatory factors IL-6 and TNF-α, thus achieving anti-inflammation and lipid reduction (58–60). Zhao et al. (61) confirmed that the amount of SCFAs was adjusted by supplementing it with dietary fiber, and, in his study, found that people with type 2 diabetes significantly increased SCFAs after consuming dietary fiber-rich foods, which were more ecologically competitive than other short-chain fatty acid-producing bacteria. Therefore, it can be concluded that dietary fiber can increase the availability of SCFAs in the body by improving the abundance of beneficial microorganisms in the gut, thus playing an important role in the body (62).

### Effect of dietary fiber on the living environment of intestinal flora

The special structure and physicochemical properties of dietary fiber positively affect intestinal health. Slow-digesting and indigestible dietary fibers cannot be hydrolyzed by human digestive enzymes to reach the large intestine directly, which reduces the postprandial blood sugar level (63). Soluble dietary fiber is sticky and can form a gel, which can delay the gastric emptying time, thus reducing cholesterol storage. Moreover, intestinal flora uses the decomposition of dietary fiber to provide energy for itself, and the acids produced and the enzymes secreted by the intestinal flora itself will also gradually erode the insoluble dietary fiber particles and destroy the apparent structure of the particles, such as particle fracture, corrosion, holes, and reduced particle size, which, based on the rough structure, can protect the beneficial intestinal bacteria caused by SCFA secretion's intestinal pH decline. The acidified intestinal environment can inhibit the growth of the pathogen; therefore, the special structure of dietary fiber contributes to the growth of probiotics to affect metabolites and probiotics.

## Future perspectives

With the progress of genomic, metabolome, and microbiome technology, research on the gut microflora-mediated bioavailability of dietary fiber has continuously deepened. The intake of high dietary fiber can not only improve the intestinal microecology but also promote the formation of a large quantity of endogenous SCFAs, which participate in the energy metabolism in the body, regulate the energy balance, and improve the function of the body. However, the effect of dietary fiber on the body is not only related to the total amount, type, and proportion of SCFAs produced by intestinal flora metabolism but also may be closely related to physiological factors, such as individual body function status, diet structure, age, and sex. These indicate that the energy metabolism used by dietary fiber for the regulation of human intestinal function and the regulation mode of the gut–brain–bacteria axis should be further analyzed. Further research on dietary fiber should be conducted used in daily life.

## Author contributions

KG drafted the manuscript. ZY collected information. TY critically revised the initial manuscript. All authors contributed to the manuscript revision and read and approved the submitted version.

## Conflict of interest

The authors declare that the research was conducted in the absence of any commercial or financial relationships that could be construed as a potential conflict of interest.

## Publisher's note

All claims expressed in this article are solely those of the authors and do not necessarily represent those of their affiliated organizations, or those of the publisher, the editors and the reviewers. Any product that may be evaluated in this article, or claim that may be made by its manufacturer, is not guaranteed or endorsed by the publisher.
